# Automatic sleep staging of single-channel EEG based on domain adversarial neural networks and domain self-attention

**DOI:** 10.3389/fnins.2023.1143495

**Published:** 2023-04-06

**Authors:** Dong-Rui Gao, Jing Li, Man-Qing Wang, Lu-Tao Wang, Yong-Qing Zhang

**Affiliations:** ^1^School of Computer Science, Chengdu University of Information Technology, Chengdu, China; ^2^School of Life Sciences and Technology, University of Electronic Science and Technology of China, Chengdu, China

**Keywords:** unsupervised domain adaptation, automatic sleep staging, EEG data, adversarial training, attention mechanism, data augment frontiers

## Abstract

The diagnosis and management of sleep problems depend heavily on sleep staging. For autonomous sleep staging, many data-driven deep learning models have been presented by trying to construct a large-labeled auxiliary sleep dataset and test it by electroencephalograms on different subjects. These approaches suffer a significant setback cause it assumes the training and test data come from the same or similar distribution. However, this is almost impossible in scenario cross-dataset due to inherent domain shift between domains. Unsupervised domain adaption was recently created to address the domain shift issue. However, only a few customized UDA solutions for sleep staging due to two limitations in previous UDA methods. First, the domain classifier does not consider boundaries between classes. Second, they depend on a shared model to align the domain that could miss the information of domains when extracting features. Given those restrictions, we present a novel UDA approach that combines category decision boundaries and domain discriminator to align the distributions of source and target domains. Also, to keep the domain-specific features, we create an unshared attention method. In addition, we investigated effective data augmentation in cross-dataset sleep scenarios. The experimental results on three datasets validate the efficacy of our approach and show that the proposed method is superior to state-of-the-art UDA methods on accuracy and MF1-Score.

## 1. Introduction

Sleep appears indispensable in all mammals, and many studies try to unravel the regularity of sleep (Harding et al., 2019; Peng et al., 2020; Bowles et al., 2022). People have been working on this research in the past decade, among which sleep staging has significantly progressed. Sleep staging contributes to detecting sleep disorders, which are usually collected through noninvasive brain-computer interface devices. It keeps track of cerebral cortex activity using a polysomnogram (PSG), a collection of bio-signals including an electrocardiogram (EEG), electromyogram, and an electroencephalogram. According to the American Sleep Society (AASM) (Iber et al., 2007), sleep is divided into three stages: wake (W), rapid eye movement (REM), and non-rapid eye movement (NREM), and N1, N2, and N3 are the three substages of NREM. In the clinical setting, expert clinicians mainly interpret manually for sleep records. It takes a lot of time and effort for professionals to check, segment, and classify each segment 8–24 h multichannel signals into continuous, fixed-length periods of 30 seconds' epoch.

In recent years, the sleep staging problem has benefited from improvements in machine learning methods (Supratak et al., 2017; Sors et al., 2018; Phan et al., 2019a; Sun et al., 2019), which processed EEG data using various network topologies and properly trained classification models to function effectively in testing. Those methods aim at automating the tedious process. However, many sleep laboratories still rely on the manual scoring of EEG data. There are two main reasons: First, automated sleep staging algorithms still require a large number of labeled data to train the models, which needs to be done manually by sleep technicians or expert clinicians. In this context, attempting to train a deep network on a large labeled source domain and transfer it to the target domain is a good compromise. Yet, this method gives lower performances than expected, which is the second question. The data produced in sleep labs and the publicly available training data differ significantly (Nasiri and Clifford, 2020; Phan et al., 2020a). It can happen for several reasons, including various measuring sites on the skull, different sampling rates of measuring instruments (Azab et al., 2018), or inherent variability between subjects. The training (source) and the test (target) distributions are different, referred to as a domain shift problem.

**Figure 1:**
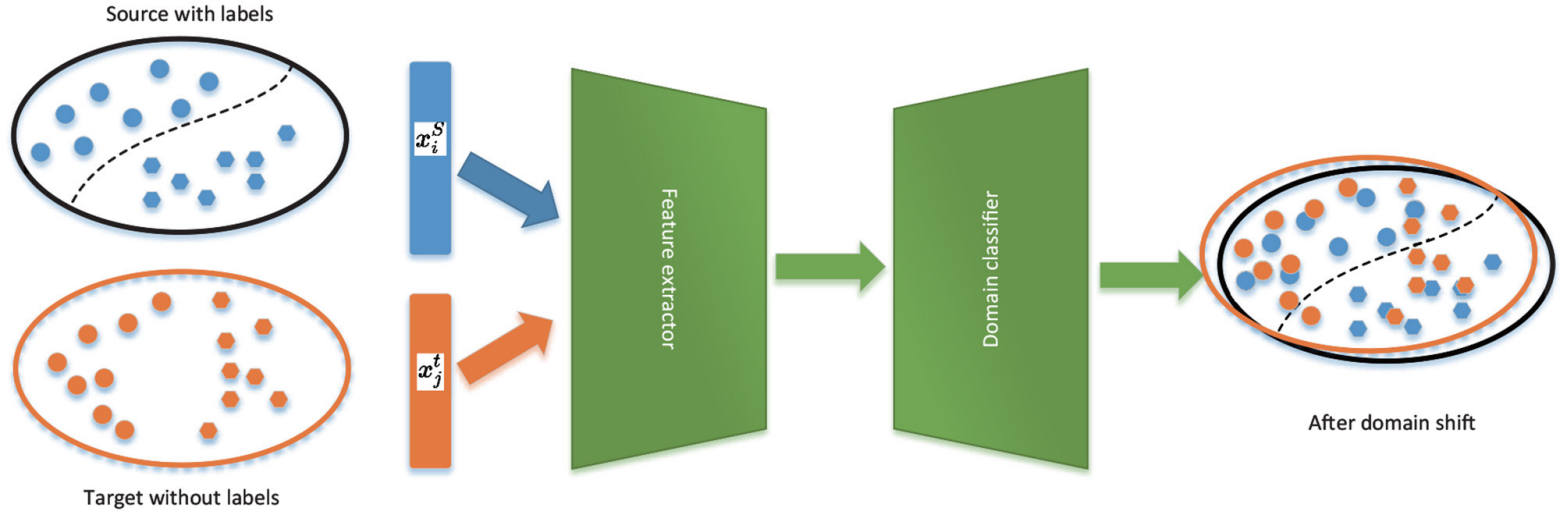
Domain adversarial neural networks.

Unsupervised domain adaptation (UDA) (Ganin and Lempitsky, 2015) has lately shown great potential in enhancing deep learning models when labeled data is scarce. It can solve the two problems above simultaneously. Firstly, it does not need mass-labeled data cause it transfers knowledge from the domain source with rich labels to the target domain with imperfect labels. Secondly, it solves the problem of distribution differences between source and target domains by aligning the distribution of source features and targets. From Figure 1, UDA uses both the labeled source domain and the unlabeled target domain to train the model to perform well on both the source and target domains. UDA is widely used in machine vision (Wang and Deng, 2018) to reduce the discrepancy between the source and the target distributions without utilizing any labels from the target domain. Some research has examined UDA's role in classifying sleep stages thus far. For instance, Chambon et al. (2018) improved the feature transferability between source and target domains using the best transport domain adaptation. Besides, Nasiri and Clifford (2020) used adversarial-based domain adaptation to increase feature transferability.

In UDA, a domain classifier (also known as a discriminator) and a feature generator are used as two players to align allocations in an adversarial way. However, applying these methods to automatic sleep staging continues to have the following drawbacks. First, they disregard the connection between the decision border and the target samples when aligning distributions. In other words, the main task of the generator is to match the distribution between the source and the target. This theory assumes that the classifier can correctly classify these target features because they are consistent with the source samples. So, they do not consider the relationship between the target sample and the decision boundary for distribution alignment. As shown on the right in Figure 1, because the generator is only attempting to make the two distributions close rather than the categorization boundary, it can yield ambiguous features close to the boundary. Secondly, they rely entirely on a common frame to extract features from the source and target domains. This could result in the loss of source and target domain-specific features, which is detrimental to classification tasks in the target domain.

To overcome the challenges above, this article presents a novel framework named Task-Domain Specific Adversarial Network (TDSAN), composed of a feature generator, a domain discriminator, and a dual classifier. It aims to align feature distributions from the source and target domains by combining the classifier's output for the target data and the domain discriminator's output for the domain identifier. Second, we create a domain-specific attention module to maintain source and target-specific features.

Specifically, we train a domain discriminator to predict the input domain and a dual classifier to predict task-specific class labels. We use domain pseudo-labels (i.e., source domain as “1” and target domain as “0”) as input. The domain discriminator is trained until it cannot distinguish between the distributions of its training and test domain examples. At the same time, the dual classifier is used to correctly classify the source samples while being trained to find target samples that are located far from the source of support. Because they are not grouped into any classes, samples far from the support do not have traits that can be used to differentiate between them. That is to say. While considering the classifier's output to the target samples, it is instructed to produce desired features close to the support points simultaneously. Therefore, our approach uses a domain discriminator to distinguish the features between samples drawn from the source domain and drawn from the target domain by predicting the domain label and a dual classifier to generate the discriminative features of the target sample because it considers how the decision boundary and the target data relate, and training is adversarial. Additionally, we use domain-specific attention to clean up the extracted features so that each domain keeps its essential characteristics.

The contributions of our paper are summarized as follows: a novel cross-dataset sleep classification framework is proposed that simultaneously changes the categorization boundaries between classes and the conditional distribution between domains. The algorithm adopts a non-shared attention module to keep critical features during adaptation, thereby improving adversarial performance on the target domain. Aiming at the data imbalance in sleep staging, we applied data augment to effectively improve the impact of sample skew on the classification network. Numerous tests show that our TDSAN delivers more excellent cross-domain sleep stage classification performance compared to cutting-edge UDA techniques.

The rest of this paper is organized as follows. Section 2 introduces related work of EEG sleep classification on domain adaptation and describes the proposed model. In Section 3, the experimental results are presented and debated. Section 4 concludes this study.

## 2. Materials and methods

### 2.1. Related work

The use of single-channel EEG for automated sleep staging has received much attention in the literature. Specifically, deep learning-based techniques (Sors et al., 2018; Kuo and Chen, 2020; Fan et al., 2021; Lee et al., 2021) have made significant progress. These approaches create various network structures to extract characteristics from EEG data and capture temporal dependencies. However, these methods often require enough labeled data to train networks with thousands of parameters. Furthermore, all the above scenarios assume that the training and test data distribution is the same or very comparable, which frequently is not the case because different psychological states or complex equipment noise may cause changes in data distribution. Although these methods have been successful in dealing with complex EEG data, they have limited results in sleep stage classification across domains (Wu et al., 2020) (e.g., cross-datasets and cross-devices) because of domain shift. As a result, numerous studies were told to use transfer learning techniques to address this problem.

There have been a few studies investigating the problem of individual sleep staging using transfer learning (Mikkelsen and De Vos, 2018; Phan et al., 2020b) to increase the specific subject's classification accuracy within a similar dataset. They exclude two nights of test subjects for datasets with two nights of recordings per subject and pretrain the model. Then, the data from the other night is used for evaluation, while the data from the first night is used to fine-tune the model.

The cross-dataset scenario, which involves training a model on data from one dataset and evaluating it on another dataset, has yet to receive much attention. Using a sizable source dataset and another labeled but small target dataset, Phan et al. (2020a) investigated the problem of data variability. Abou Jaoude et al. (2020) also applied a similar transfer learning strategy for extended scalp EEG recordings. They used the larger source dataset to train their model and the smaller target dataset to refine it. Similar to the problem scenario, Phan et al. (2019b) proposed using deep transfer learning to overcome the channel mismatch between the two domains. Abdollahpour et al. (2020) also used this idea to predict sleep staging on fused features on pretrained models. Guillot and Thorey (2021) composed eight heterogeneous sleep staging datasets into a large corpus, which solved the problem of incompatible input data shapes on tasks across datasets and improved the classification accuracy in the target domain. Moreover, even though some studies were not focused on sleep staging, they classify EEG/EMG for fatigue or motor image research and provide some practical examples. For example, Soroushmojdehi et al. (2022) proposes a subject-transfer framework. It uses the information learned from other subjects to make up for the data from the target subject. This article is about a study of hand movement intention identification based on EMG signals. Perry Fordson et al. (2022) also propose a domain adaptation method. It tries to individually treat features from auditory and visual brain regions, which successfully tackles subject-to-subject variations.

Both a labeled target dataset and a sizable corpus of source datasets are necessary for these techniques to fine-tune their models. To solve these problems, UDA strategies that align the traits from several domains with a few annotation data were presented. These methods can be classified as discrepancy-based approaches and adversarial-based approaches. Discrepancy-based methods, such as Maximum Mean Difference (MMD) (Long et al., 2015, 2016) and Correlation Alignment (CORAL) (Sun et al., 2016), strive to reduce the distance measured between the source and target distributions. On the other hand, adversarial-based methods are like Generative Adversarial Networks (GAN) (Goodfellow et al., 2014). This approach trains a domain classifier to predict the input domain and a class classifier to predict task-specific class labels. Both classifiers share the feature extraction layer. The two layers are trained to predict the labels of the source samples correctly as well as to fool the domain classifier. This method is used in current sleep staging works. It was recommended by Zhao et al. (2021) to employ adversarial UDA with a domain discriminator and several classifiers fed from different feature extractor layers. Nasiri and Clifford (2020) employed adversarial training and strategies for focusing local and global attention to extract transferable personal information. Yoo et al. (2021) used three discriminators for adversarial domain adaptation. One is the global discriminator, and two are local discriminators. The local discriminators will preserve the intrinsic structure of sleep data and reduce local misalignment. Eldele et al. (2022) used a dual classifier for the adversarial domain adaptation framework to improve the accuracy of the decision boundary.

The distribution alignment approaches based on GAN or MMD do not consider the connection between decision boundaries and target samples. Saito et al. (2018) presented an unsupervised adaptation method on the bias of Maximum Classifier Difference (MCD). The technique has a generator module to extract high-level features from the source and target domains. MCD matches distributions by producing representations within similar task-specific boundaries. Dual classifiers are proposed following the same structure. First, the annotated source data is trained to obtain two different classifiers. Second, target samples that are not supported by the source are found. Then, the L1 distance of the probability output is employed to measure the difference between the two classifiers. In this stage, the difference is maximal. It will result in the separation of the two classification boundaries. The generator will relocate the outliers within the target domain. Third, the exact differences are then minimized. Due to the two distributions widely overlapping in stage 2, the target domain is a component of the source domain in this stage. Training deals with the minmax issue by maximizing the target variance and generating a representation that minimizes features. Inspired by this, we set two discriminators. The domain discriminators for domain adaption and dual classifiers for classification boundaries adapting. The classification boundaries are between classes. A generator for generating the minimized differences features. Furthermore, we improve the adversarial training process by maintaining domain-specific features through domain-specific attention.

### 2.2. Proposed method

Section 2.2.1 briefly introduces the notations and definitions. In Section 2.2.2, we outline the structure and the details. Finally, the whole training process is described in Section 2.2.3.

The definition and notations of EEG-based sleep staging are first briefly discussed in this section. We denote $Xs={\left\{\left({\text{x}}_{s}^{i},y_{s}^{i}\right)\right\}}_{i=1}^{m_{s}}$ with *m*_*s*_ labeled source data and $X_{t}={\left\{\left({\text{x}}_{t}^{i},\right)\right\}}_{i=1}^{m_{s}}$ with *m*_*t*_ unlabeled samples. In the context of EEG data, $x_{s}^{i}$ and $x_{t}^{j}\in R^{1*T}$, since EEG data is 2D time series data, symbol 1 means channels (electrodes) and *T* means how many numbers of timesteps are in each 30-second EEG epochs. Feature extractor *F*, receives *X*_*s*_ or *X*_*t*_ as input. Domain attention module *A* receives the output of the feature generator, dual classifier networks *C*1, *C*2, and domain classifier *D* extract features from *A* and classify them. The dual classifier networks classify the extracted features into *K* classes, i.e., output a 5-dimensional logarithmic vector. The domain classifier classifies them into two classes, which are set to be one if the data come from the source domain and set to 0 otherwise. All the class probabilities are obtained through the softmax function. Here, the softmax function's activation of the L1 distance between the probabilities of the two classifiers serves as the discrepancy loss. Following the experience of Saito et al. (2018), we denote the discrepancy loss as follows:


(1) $$\begin{array}{l} d\left(p_{1},p_{2}\right)=\frac{1}{K}\overset{K}{\underset{k=1}{\sum }}|p_{1k}-p_{2k}| \end{array}$$

where *p*_1_*k* and *p*_2_*k* represent the probability outputs of *p*_1_ and *p*_2_ of *k* classes, respectively. We also have an adversarial loss, which tries to deceive the domain classifier by confusing the two data domains.

We aim to acquire a feature generator that minimizes the variance of the target samples.

#### 2.2.1. Network framework

**Figure 2:**
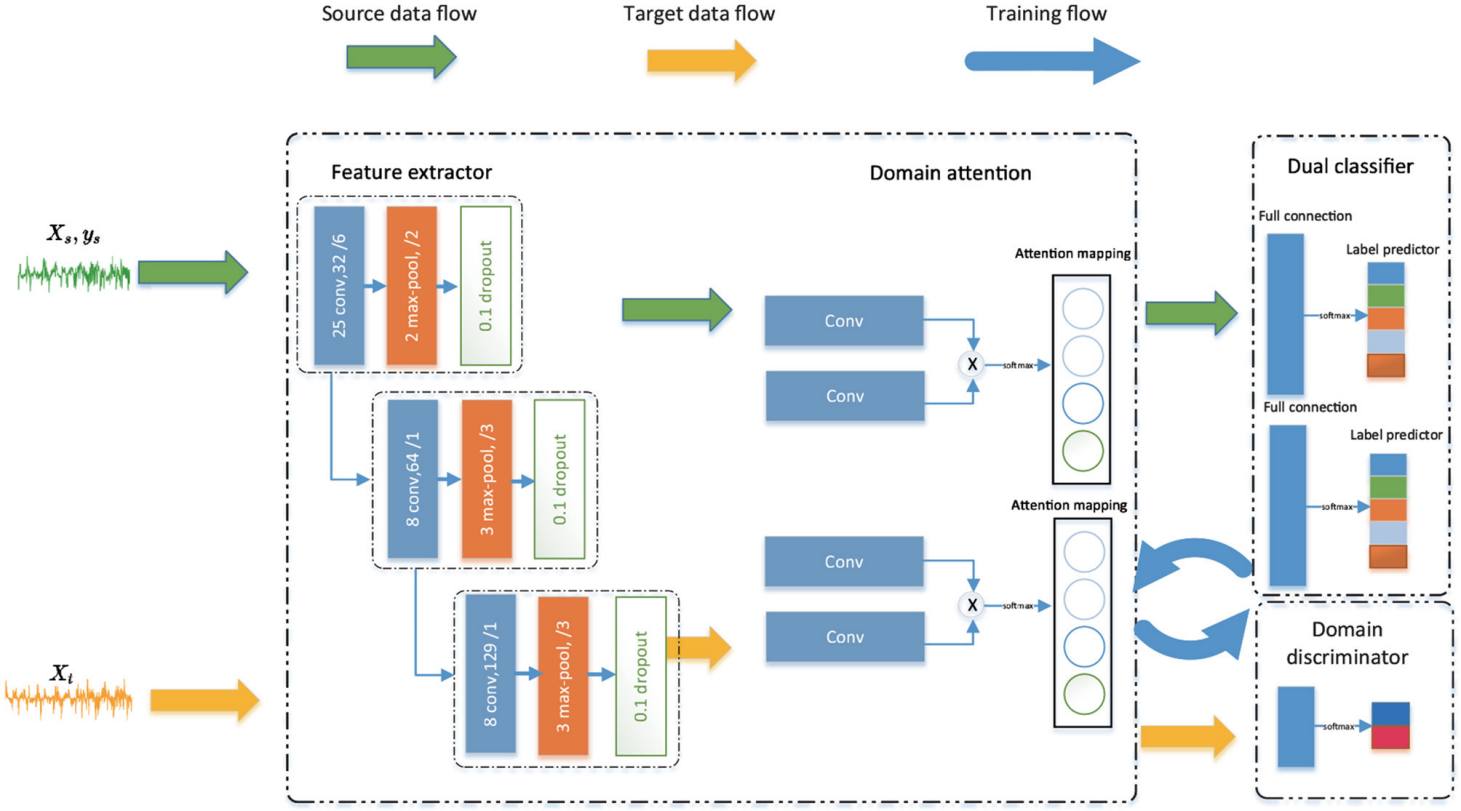
Overall architecture of the proposed TDSAN framework.

We propose the TDSAN model, which consists of a feature extractor, a domain-specific attention module, a dual classifier, and a domain classifier. The overall model of TDSAN is shown in Figure 2. We first extract the shared feature to generate high-level features representation using both source data and target data. Then, domain-specific attention is put in charge of calculating the relevance of the time sequence. It plays a crucial role in keeping each domain's useful features by fine-tuning the extracted features. The feature extractor and the domain-specific attention together form a generator module. The dual classifier has the following two functions. First, complete the classification task. Second, it is iteratively trained with the generator as a discriminator. It tries to align the distribution on task-specific boundaries. The domain classifier recognizes the domain ID to align domain feature distribution.

##### 2.2.1.1. Feature extractor

The feature extractor consists of three CNN-based convolution blocks, each in 1 dimension. To speed up training and keep CNN Network stable, we use a batch normalization layer. It can reduce internal covariate shift (Perry Fordson et al., 2022). We use leaky ReLU as an activation function and MaxPooling to reduce information redundancy. Given an input source sample *x* ∈ *R*^1·*T*^, it generates source features through a feature extractor, that is, *F*(*x*) = (*fl*, …, *fl*) ∈ *R*^1·*l*^ where *l* is the length of the feature.

##### 2.2.1.2. Domain-specific attention

After convolution, EEG data output is shorter 2D EEG data usually contains the temporal dimension. We do not simply treat the EEG series as a particular image. Based on such consideration, we attempt to extract recessive temporal features from EEG using temporal self-attention mechanisms. In addition, we get shared features after feature extraction. However, different datasets may have different temporal features. Therefore, we use a non-shared attention module to extract domain-specific information. Extracting different domain time temporal also plays a vital role in fine-tuning features. The attention module computes a weighted sum of the features at all locations with the bit of computational cost for each location in the feature space. As a result, each site's features contain intricate details that correspond to fine details in the feature's distant sections.

Inspired by Zhang et al. (2019), as seen in Figure 2, we use a convolutional attention method. The attention operation obtains a feature representation at each location based on two 1D convolutions (e.g., *M*_1_ and *M*_2_). Particularly, ${\text{p}}_{sx},{\text{p}}_{sy}\in {\mathbb{R}}^{d}$ are the eigenvalues of positions *x* and *y*, which are transferred into *Q*_*sx*_ = *M*_1_(**p**_*sx*_) and *Q*_*sy*_ = *M*_2_(**p**_*sy*_). Note that the score is expressed as:


(2) $$\begin{array}{l} {𝒲}_{yx}=\frac{\exp\left(Q_{sx}^{⊤}Q_{sy}\right)}{\overset{l}{\underset{k=1}{\sum }}\exp\left(Q_{sk}^{⊤}Q_{sy}\right)} \end{array}$$

where 𝒲 _*xy*_ represents the degree to which the *y*-*th* location pays attention to the *x*-*th* position. The output is


(3) $$\begin{array}{l} {\text{o}}_{sy}=\overset{l}{\underset{x=1}{\sum }}{𝒲}_{yx}{\text{p}}_{sx} \end{array}$$

Equations 1 and 2 refer to the attention process as *A*(·); therefore, *O*_*s*_ = *A*_*s*_(*F*(*x*_*s*_)). The same procedure applies to the target domain data stream to train *A*_*t*_.

##### 2.2.1.3. Dual classifier

The dual classifier consists of three fully connected layers. A dual classifier has two tasks: acting as a classifier for the source data and detecting the target samples outside the source support when we combine two category classifiers. The dual classifiers C1 and C2 first try to maximize the difference for a given target feature to find the target samples away from classification boundaries. Then it minimizes the discrepancy to make them close to the classification boundary. By iterating this process repeatedly, we train a generator that has rare differences with the classification Boundary of the source domain.

##### 2.2.1.4. Domain discriminator

It is widely used in UDA (Tzeng et al., 2017). The main goal is to minimize and regularize the distance between the empirical mapping distributions by training iteratively with the generator. Specifically, We first introduce the domain discriminator by using source samples and target samples with their domain labels. Subsequently, we fix the domain discriminator and train the generator to deceive the discriminator confusing the data domain. We finally get the domain-invariant representations through iterative training.

The details of these four modules are shown in Table 1.

**Table 1:** Model parameters of the feature extractor and classifiers.

**Modules**	**Layers**	**Kernel**
Feature extractor	Conv1d	1x25,32
	BatchNorm1d	
	MaxPool1d	1*2,stride 2
	Conv1d	1x8,64
	BatchNorm1d	
	MaxPool1d	1*3,stride 3
	Conv1d	1x8,128
	BatchNorm1d	
	MaxPool1d	1*3,stride 3
Domain attention	/	/
Dual classifier	FC	1,024
	FC	512
	FC	5
Domain discriminator	FC	1,024
	FC	512
	FC	2

#### 2.2.2. Training steps and loss function

We described the four modules in detail in Section 2.2.1. This section describes the training steps and loss functions to train the entire network. First, the framework of the overall network is to solve a maximize problem. It should be noted that the training is iterative between the generator and the dual classifier and between the generator and domain Discriminator. We solve this problem in four steps.

##### 2.2.2.1. Train the dual classifier

First, the CNN and the attention mechanism network extract the high-level representation. Then we put them in the dual classifier and train it to get two different decision boundaries with source data. We use cross-entropy loss since the data is labeled. It should be noted that C1 and C2 are initialized differently to obtain two different decision boundaries.


(4) $$\begin{array}{l} \underset{G,c_{1},c_{2}}{\min}L\left(X_{s},Y_{s}\right) \end{array}$$

(5) $$\begin{array}{l} L\left(X_{s},Y_{s}\right)=-E_{\left({\text{x}}_{s},y_{s}\right)\sim \left(X_{s},Y_{s}\right)}\overset{K}{\underset{k=1}{\sum }}I_{\left[k=y_{s}\right]}\log p\left(y|O\left(x_{s}\right)\right) \end{array}$$

##### 2.2.2.2. Fixed generator, training domain discriminator

In this step, the source domain ID is one, and the target domain ID is 0. It aims to minimize the domain confusion loss for source and target samples on a fixed *G*. This is a supervised learning process.


(6) $$\begin{array}{l} L=-\overset{N}{\underset{i=1}{\sum }}y^{\left(i\right)}\log{ŷ}^{\left(i\right)}+\left(1-y^{\left(i\right)}\right)\log\left(1-{ŷ}^{\left(i\right)}\right) \end{array}$$

##### 2.2.2.3. Fixed generator, dual training classifier

In this step, we train classifiers *C*1 and *C*2 to minimize discrepancy on target samples for a fixed generator *G*. They can detect target samples far from source support and force them to relocate to the corresponding category.


(7) $$\begin{array}{l} L_{\text{adv}}\left(X_{t}\right)=E_{{\text{x}}_{\text{t}}\sim X_{\text{t}}}\left[d\left(p_{1}\left(\text{y}|O\left({\text{x}}_{\text{t}}\right)\right),p_{2}\left(\text{y}|O\left({\text{x}}_{\text{t}}\right)\right)\right)\right] \end{array}$$

##### 2.2.2.4. Fixed dual classifier and domain discriminator, training generator

In this step, we train generator *G* to maximize discrepancy on target samples. It identifies the target samples that the source's support has eliminated.


(8) $$\begin{array}{l} \underset{c1,c2}{\max}L_{\text{adv}}\left(X_{t}\right) \end{array}$$

In our system, these three phases are repeated. Our primary focus is on adversarially training classifiers and generators to identify source samples and confusing domain distribution. Algorithm 1 summarizes the complex algorithm of TDSAN.

**Algorithm 1:** Training Procedure for TDSAN

**Input**: source data *Xs, Ys*, target data *Xt*
**for** *epoch in maxepoch* **do**
**for** *each mini-batch* **do**
pretrain *C*1, *C*2 with source *Xs, Ys*
reduce 𝒪(*f*_*s*_) and 𝒪(*f*_*t*_)
**for** *p* *in* *F)* **do**
train *D* for 𝒪(*f*_*s*_) and 𝒪(*f*_*t*_)
**end**
train *C*1, *C*2 with source *Xs, Ys*
maximize discrepancy using the class
probability of target *f*_*t*_
reduce task-specific variance based on the class
probability of target *f*_*t*_
**end**
**end**

## 3. Results

### 3.1. Data

We evaluated the proposed framework on three datasets, including two public and one private dataset, namely sleep-EDF-SC (EDF), sleep-EDF-ST(ST), and self-collection datasets. Before downsampling, a summary of the three datasets above is displayed in Table 2.

**Table 2:** A brief description about the datasets.

**Dataset**	**Subject**	**Recordings**	**Sample rate**	**Channel**	**Scoring**
EDF	20	39	100	Fpz-Cz	R&K
ST	22	44	100	Fpz-Cz	R&K
self-collection	6	30	250	C4-A1	AASM

#### 3.1.1. Public datasets

The sleep-EDFx dataset (Goldberger et al., 2000) is made up of 42 subjects' 61 polysomnographic (PSG) data and corresponding hypnograms (annotations by sleep experts). European data format (EDF) is used to store PSG records, while EDF+ is used to store hypnograms. A horizontal EOG channel, a sub-mental chin EMG, two EEG channels, Fpz-Cz and Pz-Oz, an event marker, and an EOG channel are all included in each record. At 100 Hz, the EEG signal is captured. The annotations for each stage of sleep in the hypnogram file are W (wake), R (REM), 1 (N1), 2 (N2), 3 (N3), 4 (N4), M (movement time), and not scored (denoted as?). 42 participants from two separate groups—the Sleep Cassette (SC) group and the Sleep Telemetry (ST) group—were employed in the study. The ST group consists of the remaining 22 participants with modest sleep problems. In contrast, the SC group consists of the remaining 20 healthy subjects who are not taking any medicine. Every SC subject in the EDFx dataset has two nights' sleep records, except one, which only has one. 22 sick participants were recorded for one night of sleep by the ST group. Based on the experience of Supratak et al. (2017) and Eldele et al. (2021), we evaluate our model on the channel Fpz-Cz.

#### 3.1.2. Self-collection datasets

This study conducted 30 nights of polysomnography trials following clinical recommendations and the AASM guideline. There are six subjects, each under observation for five nights. The subjects were all young, healthy adults between the ages of 20 and 24, with a male-to-female ratio of 1:0.67. Each participant voluntarily agreed to take part in this sleep study. They had to bathe and do their hair before the investigation to keep their heads tidy. The time ranged from 23:00 to 07:30, exceeding 8 h. All participants ensured they were in good health, had no medical history, and hadn't done any strenuous exercise the hour before the sleep experiment began. The 3-channel EEG, 2-channel EOG, 1-channel EMG, and 1-channel ECG signals were accurately acquired and stored. The system gain is 24, and the sampling rate is 250 Hz. In addition, conductive gel paste is applied to all the gold-plated disc electrodes used in EEG electrodes for signal acquisition. Patch electrodes are used in EOG, EMG, and ECG electrodes. The subject's dormitory bed served as the site for the entire sleep experiment, and the AASM suggested all electrode implantation settings. Three EEG channels, i.e., C4-A1, F4-A1, and O2-A1, were combined to form the electrode title, which is now only C4, F4, and O2. Then, EOG-R and EOG-L are combined to form REOG and LEOG from the two EOG channels. We evaluated our model on channel C4-A1.

### 3.2. Data preprocessing

In the 30 s/epoch time series, the filtered data were separated into non-overlapping pieces according to the AASM staging criteria.

- Epochs classified as being in motion, artifacts, or unknown were eliminated.
- To meet the AASM norm, sleep stages S3 and S4 were combined into a single N3 stage.
- Only the first and last 30 mins of wake time were included.
- Downsampling the data with a sampling frequency higher than 100 Hz, and the length of a single epoch is 30 s × 100 Hz (T = 3,000).
- Cutoff frequency design: based on high-pass and low-pass filters (0.3-35 Hz) to reduce the noise.

### 3.3. Experimental settings

We used the macro-averaged F1-score and the classification accuracy (ACC) to assess the proposed performance. The metrics are denoted as follows:


(9) $$\begin{array}{l} ACC=\frac{\overset{K}{\underset{i=1}{\sum }}TP_{i}}{M} \end{array}$$

(10) $$\begin{array}{l} MF1=\frac{1}{K}\overset{K}{\underset{i=1}{\sum }}\frac{2\times {\text{Precision}}_{i}\times {\text{Recall}}_{i}}{{\text{Precision}}_{i}+{\text{Recall}}_{i}} \end{array}$$

where $\mathrm{Precision}=\frac{TP}{TP+FP}$, $\mathrm{Recall}=\frac{TP}{TP+FN}$. TP, FP, TN, and FN denote True Positive, False Positive, True Negative, and False Negative, respectively. The whole sample number is *M*, and the total class number is *K*. The experiment was model initialized from various random seeds and repeated five times. The average final result (ACC and MF1) was then presented with the standard deviation.

We divide the experimental data into 80% and 20% for training and testing. We do not disrupt the order of epochs of subjects so that domain-attention-specific can capture the relationship between different sleep stages. We employ the Adam optimizer with a batch size of 128 and a learning rate of 1e−3. We did not fine-tune these hyperparameters for a fair comparison. Another hyperparameter is n, which represents the number of times this operation is repeated for the same mini-batch. This value represents the trade-off between the generator and the classifier. All experiments are done by pytorch1.12 on an NVIDIA GeForce RTX 2080 Ti GPU.

### 3.4. Baselines

We analyze our suggested TDSAN by contrasting it with different baselines. We started by including the Direct Transfer (DT) findings from DeepSleepNet's (Supratak et al., 2017) three sleep staging methods. In addition, we adopted four state-of-the-art baselines based on adversarial domain adaptation (DA). We briefly describe these baselines:

- DANN (Eldele et al., 2021): It simultaneously trains a feature extractor and a domain classifier using a gradient reversal layer (GRL) to remove the gradient of the domain classifier.
- ADDA (Ganin et al., 2016): It accomplishes a comparable task to DANN but reverses the labels.
- CDAN (Tzeng et al., 2017): Minimize the cross-covariance between feature representations and classifier predictions.
- ADAST (Yoo et al., 2021): It uses dual classification on top of domain obfuscation to consider class-conditional distributions.

### 3.5. Results

Tables 3, 4 show the comparison results among various methods. It suggested that the direct transfer results are usually the worst. This result indicated that the domain shift issue has a significant impact and needs to be handled independently. The findings of the other 4 DA baselines confirm the need for domain adaptation to overcome the domain shift issue.

**Table 3:** Comparison of different baselines on ACC. Bold: the best results; Underlined: the second best results.

	**Baselines**	**EDF → ST**	**EDF → SC**	**ST → EDF**	**ST → SC**	**SC → EDF**	**SC → ST**	**ACC**
DT	DeepSleepNet	72.34	61.53	68.35	50.23	62.75	49.85	60.84
DA	DANN	73.70	65.98	64.23	58.94	65.93	67.53	66.05
	ADDA	73.72	77.40	64.14	65.93	58.43	68.65	68.71
	CDAN	**76.42**	67.36	66.68	70.36	62.89	**70.06**	68.96
	ADAST	72.85	70.41	71.23	70.06	**68.41**	69.88	70.47
	TDSAN(ours)	73.97	**79.32**	**73.90**	**70.89**	65.12	68.19	**71.89**

**Table 4:** Comparison of different baselines on MF1. Bold: the best results; Underlined: the second best results.

	**Baselines**	**EDF → ST**	**EDF → SC**	**ST → EDF**	**ST → SC**	**SC → EDF**	**SC → ST**	**MF1**
DT	DeepSleepNet	56.58	45.88	53.86	44.83	55.32	40.30	50.12
DA	DANN	61.35	55.79	53.49	50.63	55.19	56.63	55.51
	ADDA	59.69	55.5	55.69	54.79	48.89	**63.86**	56.40
	CDAN	**63.06**	54.73	53.91	64.01	52.61	59.71	57.95
	ADAST	59.86	**61.89**	**60.72**	**64.29**	**56.65**	60.33	**60.62**
	TDSAN (ours)	60.00	60.66	59.91	58.95	53.29	55.30	58.01

It is important to emphasize that we use the proposed backbone feature extractor on four baseline methods except DeepSleepNet to ensure a fair comparison. In this setting, we note that methods that consider class-conditional distributions: such as CDAN and ADAST, outperform the globally aligned source and target domains, namely DANN and ADDA. This shows that taking class distribution into account is crucial to improving classification performance on the target domain, mainly when dealing with imbalanced sleep data. Our proposed TDSAN outperforms all baselines in accuracy in four of the six cross-domain scenarios and achieves the second-best average score among all baselines on *MF1*-*Score*.

We consider possible reasons: First, by iteratively training the generator and classifier, we obtain a feature extractor that can extract the smallest difference between the source and target domains. At the same time, the task-specific classifier fully considers the relationship between the target task and the decision boundary. Second, performance is enhanced by TDSAN because it uses a non-shared attention module to preserve domain-specific features.

In Tables 3, 4, we also find essential clues on different cross-dataset situations. In SC → EDF and SC → ST, they are generally lower than other scenarios in various cross-domain scenarios. This may be because the dataset is too small, and the classification performance of the classifier on the source domain data is poor, which is insufficient to correct the do-main offset results. In addition, we also observed that the transfer results of datasets between SC and ST are relatively poor. We observed from the characteristics of datasets that EDF and SC datasets are the sleep EEG data of healthy people, while ST data sets are collected from people with mild sleep disorders. In addition, the acquisition channels and frequencies of ST and SC are different, so they are a relatively remote domain from EDF and SC. These findings suggest that adapting to distant domains is still exceedingly difficult.

### 3.6. Data augmentation

**Figure 3:**
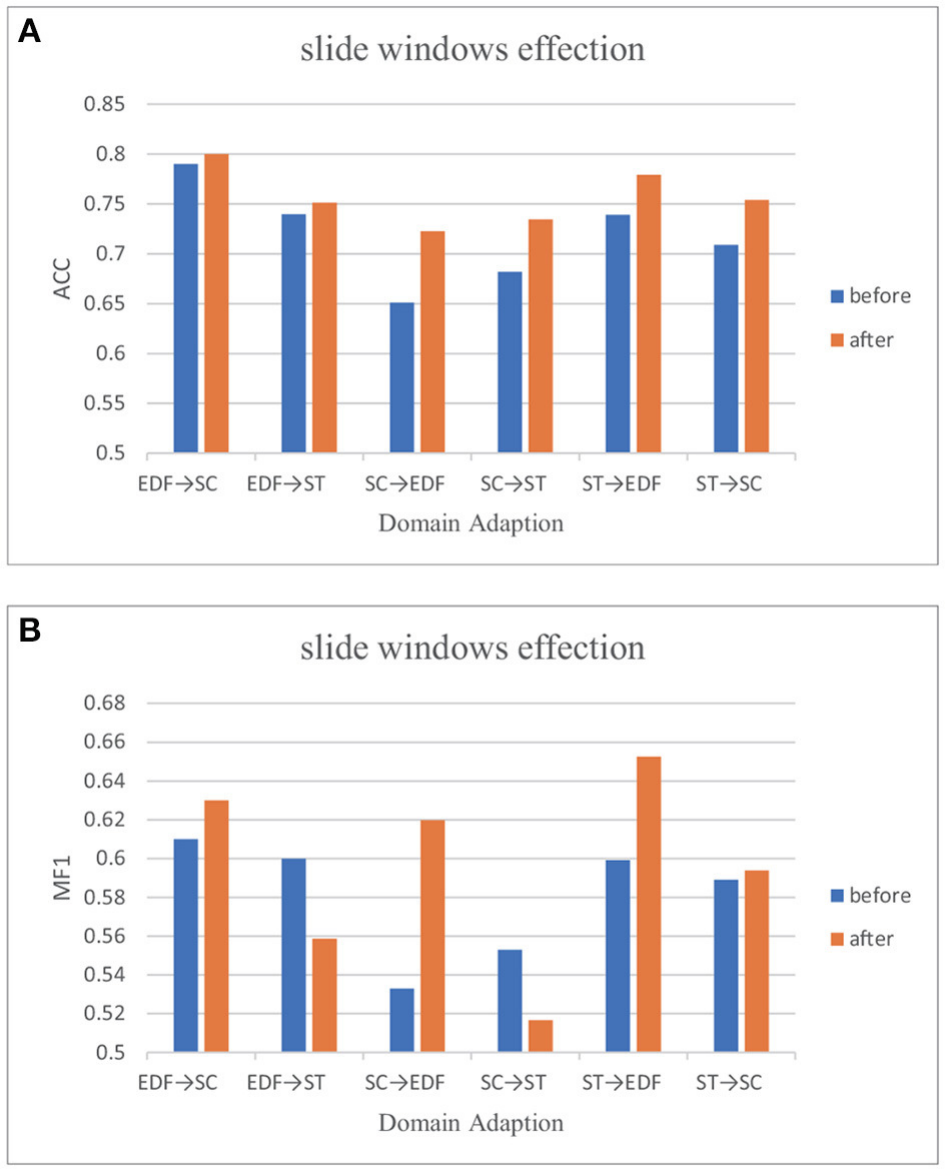
Classification performance before and after data augment. **(A)** Performances on ACC before and after sliding window. **(B)** Performances on MF1 before and after sliding window.

Each stage's length varies for a sleep recording. Mainly, stage N2 makes up between 45% and 55% of the entire sleep time and contributes to the majority class. N1, on the other hand, only makes up roughly 2%–5% [36]. Every sleep dataset that is accessible has this problem, including public datasets we can retrieve and our self-collection dataset. As mentioned in many studies (Tsinalis et al., 2016a,b; Supratak et al., 2017; Sun et al., 2019), class imbalance may hinder the classifier's performance, limiting the improvement of automatic sleep staging algorithms. Following the suggestion of Ko et al. (2021), we use the sliding window method to augment the N1 stage data. The window size is 30s, and the step size is 25s. We are also experimenting with six cross-domain scenarios to see if augmentation affects the classification results. Table 5 shows the comparison before and after data augmentation on six cross-domain scenarios with three indexes, precision, recall, and MF1_score in detail. For easier reading, we use Figure 3 to present these results.

**Table 5:** Classification performance before and after data augment.

**Cross-domain**	**Slide windows**	**Sleep score**	**W (%)**	**N1 (%)**	**N2 (%)**	**N3 (%)**	**REM (%)**	**ACC (%)**	**MF1 (%)**
EDF → ST	Before	Precision	50.00	34.42	84.24	97.26	66.55	73.97	66.49
		Recall	71.95	43.44	81.83	27.95	88.94	73.97	62.82
		F1-score	59.00	38.41	83.02	43.43	76.13	73.97	60.00
		Support	82	122	1156	254	434	73.97	2048
	After	Precision	80.17	60.00	94.36	96.19	61.70	75.12	68.10
		Recall	96.04	28.68	95.81	55.04	73.32	75.12	53.03
		F1-score	87.39	38.81	95.08	70.02	67	75.12	55.87
		Support	82	205	1156	254	434	75.12	2131
EDF → SC	Before	Precision	37.25	15.79	87.32	92.53	62.46	79.32	59.07
		Recall	66.67	11.11	84.22	82.29	78.60	79.32	64.58
		F1-score	47.80	13.04	85.74	87.11	69.61	79.32	60.66
		Support	323	191	925	201	408	79.32	2048
	After	Precision	56.06	22.22	85.55	91.55	61.08	80.26	63.30
		Recall	64.91	7.41	86.98	78.47	83.39	80.26	64.23
		F1-score	60.16	11.11	86.26	84.51	70.51	80.26	62.51
		Support	323	215	925	201	408	80.26	2072
ST → EDF	Before	Precision	62.18	38.24	88.77	68.77	58.63	73.90	64.92
		Recall	30.03	6.81	86.27	97.51	85.29	73.90	61.18
		F1-score	40.50	11.56	87.50	80.66	67.33	73.90	59.91
		Support	101	77	1170	36	517	73.90	1901
	After	Precision	90.67	40.63	83.95	82.99	64.03	77.94	72.45
		Recall	54.40	12.68	91.71	78.74	78.34	77.94	63.17
		F1-score	68.00	19.33	87.66	80.81	70.47	77.94	65.25
		Support	101	136	1170	36	517	77.94	1960
ST → SC	Before	Precision	62.18	38.24	88.77	68.77	61.63	70.90	61.92
		Recall	30.03	6.81	86.27	97.51	85.29	70.90	61.18
		F1-score	40.50	11.56	87.50	80.66	64.33	70.90	58.91
		Support	323	191	925	201	408	70.90	2048
	After	Precision	55.93	47.83	83.86	98.85	47.87	75.42	64.25
		Recall	76.74	8.21	91.21	80.54	94.07	75.42	60.83
		F1-score	64.71	14.01	86.37	88.32	62.31	75.42	58.39
		Support	323	215	925	201	408	75.42	2072
SC → EDF	Before	Precision	100.00	0.00	77.22	96.15	37.27	65.12	62.13
		Recall	19.02	0.00	86.52	87.04	97.35	65.12	56.99
		F1-score	30.59	0.00	81.60	91.37	53.91	65.12	53.29
		Support	101	77	1170	36	517	65.12	1901
	After	Precision	97.59	4.35	80.87	96.15	49.28	72.28	65.65
		Recall	43.67	1.95	92.30	91.88	90.00	72.28	63.96
		F1-score	60.34	2.70	86.21	93.97	63.68	72.28	61.38
		Support	101	136	1170	36	517	72.28	1960
SC → ST	Before	Precision	98.70	0.00	79.69	98.37	45.80	68.19	63.51
		Recall	35.49	0.00	81.22	81.82	99.12	68.19	56.53
		F1-score	37.93	0.00	80.45	89.33	58.80	68.19	55.30
		Support	82	122	1156	254	434	68.19	2048
	After	Precision	81.82	0.00	92.94	47.39	68.54	73.44	58.14
		Recall	21.95	0.00	72.84	100.00	89.86	73.44	56.93
		F1-score	34.62	0.00	81.67	64.30	77.77	73.44	51.67
		Support	82	205	1156	254	434	73.44	2131

The result suggested sample skew affects the performance of the classification network. In domain adaption, data augmentation is still optional when we can not get enough EEG data.

### 3.7. Feature visualization

Further, we use UMAP to visualize the feature representations learned to make the Comparison more intuitive.

**Figure 4:**
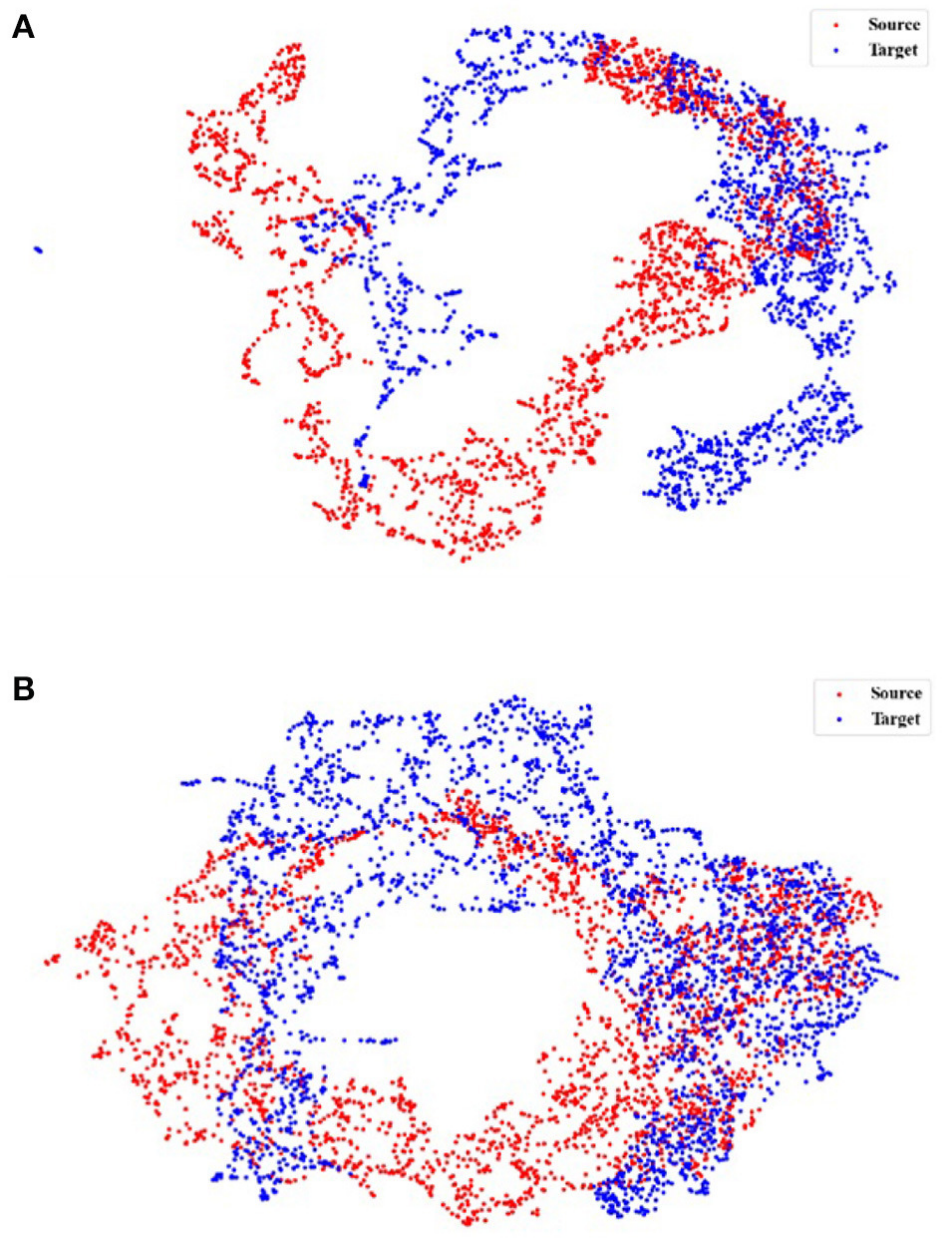
UMAP feature space visualization showing the source and target domains alignment using, applied for the scenario EDF → ST. **(A)** Before TDSAN. **(B)** After TDSAN.

Initially, we investigate the alignment quality. Figure 4 shows the alignment between the source and target domains in the EDF → ST scenario, where Figure 4A shows the feature distribution of the source and target domains before training. Our TDSAN framework alignment is shown in Figure 4B. The blue dots in these pictures offer the target domain, whereas the red dots show the source domain. The source and target domain feature distributions have significant differences before alignment. After alignment, the feature overlap between the source and target domains increases.

**Figure 5:**
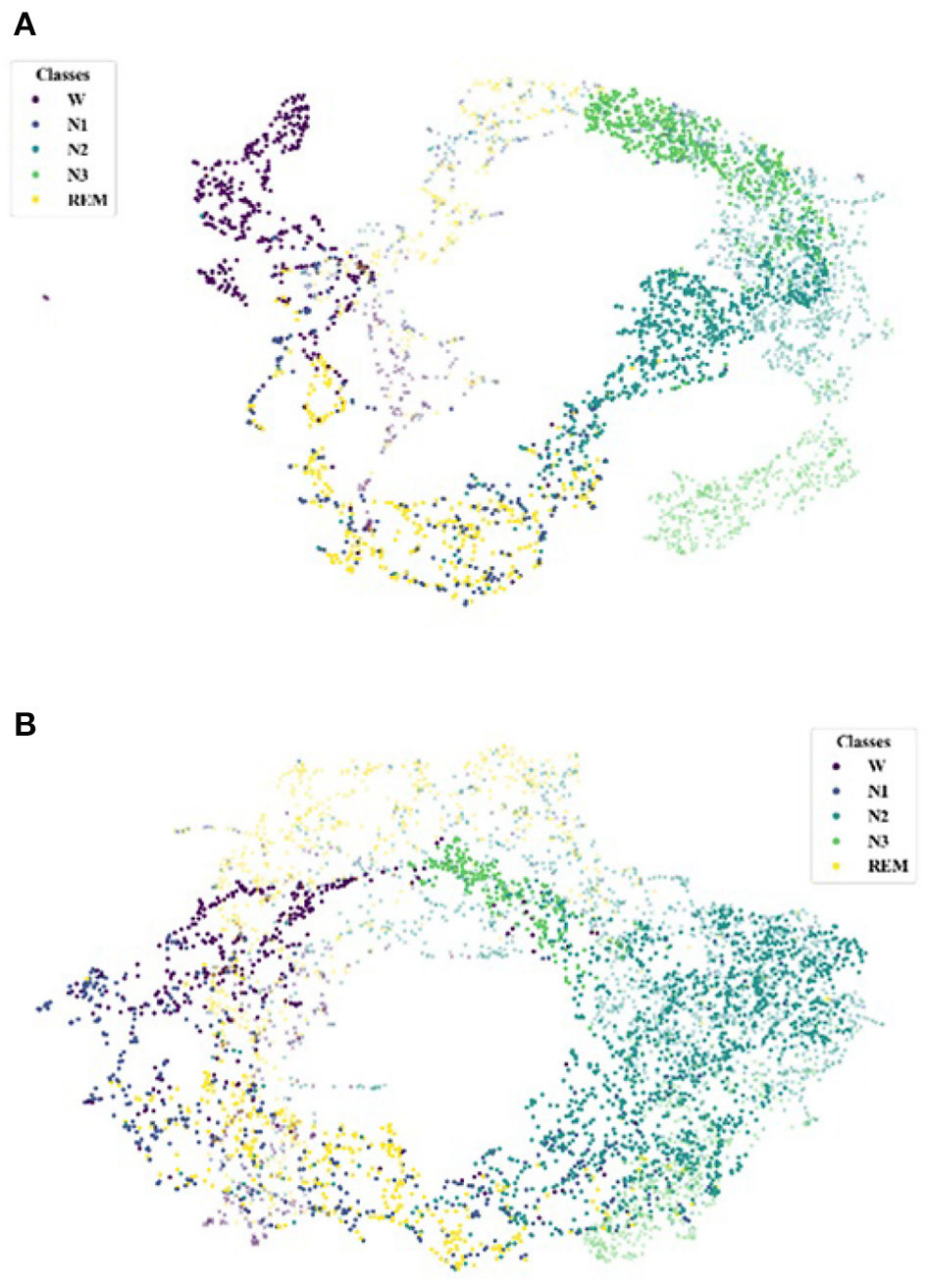
UMAP feature space visualization showing the target domains classification performance, applied for the scenario EDF → ST. **(A)** Before TDSAN. **(B)** After TDSAN.

Additionally, after the alignment in Figure 4, we investigate the target domain classification performance under the above scenarios. In particular, Figure 5A is the class distribution of the source and target domains before training, and Figure 5B is the distribution after our alignment. The symbol (·) represents the classification of the source domain data, and the emblem ( × ) represents the classification of the target domain. We note that Figure 5A shows that the type of source and target domains differs considerably. And after training, the classification of the source and target domains is much higher. This is achieved through an iterative self-training strategy.

## 4. Conclusion

This paper proposes a novel UDA method, TDSAN, to address the sleep EEG staging scores on unlabeled data. TDSAN is an adversarial learning method that uses a specific classifier as a discriminator whose target samples are remote from the source support detected. To trick the classifier, the feature generator masters to produce target features close to supports. The generator will prevent creating target features close to class boundaries since it incorporates feedback from task-specific classifiers. Meanwhile, a non-shared attention mechanism preserves domain-specific features, which can capture the relationship between different sleep stages. Experiments show that we can achieve the same accuracy on unlabeled sleep data as on labeled data.

## Data availability statement

The original contributions presented in the study are included in the article/supplementary material, further inquiries can be directed to the corresponding author.

## Author contributions

D-RG and JL: conceptualization and writing—review and editing. JL: methodology, investigation, data curation, and writing—original draft preparation. D-RG, JL, and M-QW: visualization and validation. L-TW: formal analysis and resources. Y-QZ: supervision and writing—review and editing. All authors have read and approved the final draft of the manuscript.
